# KPC Type β-Lactamase, Rural Pennsylvania

**DOI:** 10.3201/eid1210.060297

**Published:** 2006-10

**Authors:** Jonathan Pope, Jennifer Adams, Yohei Doi, Dora Szabo, David L. Paterson

**Affiliations:** * Dubois Regional Medical Center, Dubois, Pennsylvania, USA;; † University of Pittsburgh Medical Center, Pittsburgh, Pennsylvania, USA;; ‡ Semmelweis University, Budapest, Hungary

**Keywords:** KPC, extended-spectrum β-lactamase, carbapenemase, letter

**To the Editor:** Rural counties have been defined as those lacking a metropolitan center that has a population >50,000 persons (*1*). Little is known about antimicrobial drug resistance in such communities in the United States. Stevenson and colleagues (*2*) recently evaluated antimicrobial drug–resistant gram-positive infections in rural hospitals in Idaho and Utah. These researchers found that both methicillin-resistant *Staphylococcus aureus* (MRSA) and vancomycin-resistant enterococci occurred in such settings, although some of the MRSA strains were probably community associated. Comparable studies on multidrug-resistant gram-negative infections have not been performed, to our knowledge.

*Klebsiella pneumoniae* producing a broad-spectrum β-lactamase, KPC, has been described in tertiary care centers and other metropolitan hospitals in New York City. Examples have also been found in similar settings in Boston, New Jersey, Maryland, and North Carolina (*3**–**5*). The carbapenems (such as imipenem and meropenem) are typically the most active antimicrobial agents against the *Enterobacteriaceae*. The KPC β-lactamases inactivate carbapenems and all other β-lactam antimicrobial drugs. Unfortunately, bacteria producing the KPC type β-lactamases are typically also resistant to trimethoprim/sulfamethoxazole, quinolones, and aminoglycosides, thereby making these pathogens truly multidrug resistant.

We describe a patient with KPC-producing *K. pneumoniae* in a rural setting in central-west Pennsylvania. The case highlights the potential for multidrug-resistant gram-negative organisms to occur outside their previously recognized settings in large metropolitan centers.

The patient was a 76-year-old woman who lived alone, closely attended by her daughter, in a small, central Pennsylvania community, 95 miles from a metropolitan center with a population of >50,000. Her medical history included a seizure disorder, hypertension, osteoarthritis of the knees, obesity, osteoporosis, and total hysterectomy. A month before isolation of the KPC-producing *K. pneumoniae*, the patient had a 3-day hospital admission to a 200-bed hospital in the nearest metropolitan center (population 7,000) after a fall. She was discharged to a local nursing home for rehabilitation. She is not known to have visited or been hospitalized in New York, Philadelphia, or New Jersey, nor did she share a room with a patient known to have been hospitalized in these areas. She had no known animal contact. She had received trimethoprim/sulfamethoxazole and levofloxacin for treatment for urinary tract infections in the month before the KPC-producing strain was isolated. She was readmitted to the 200-bed hospital with pyelonephritis in August 2005. Cultures of urine grew *K. pneumoniae*; the organism was resistant to all β-lactam antimicrobial drugs tested, including cefepime, ceftriaxone, piperacillin/tazobactam, imipenem, fluoroquinolones, trimethoprim/sulfamethoxazole, gentamicin, and tobramycin. The patient received therapy with amikacin in combination with cefepime, ertapenem, or tigecycline at different times over the following 4 weeks. Her symptoms improved, although her urine remained colonized with the multidrug-resistant *K. pneumoniae*. In October 2005, *Clostridium difficile* infection developed, accompanied by deep venous thrombosis and gastrointestinal bleeding, and the patient died. Multiple blood cultures collected before her death were negative, although the urine was persistently colonized with the multidrug-resistant *K. pneumoniae*.

The organism was referred to a research laboratory in a metropolitan center ≈100 miles away. Molecular analysis of the mechanisms of resistance was performed by using previously described methods (*6*). This analysis showed that the *K. pneumoniae* isolate produced the extended-spectrum β-lactamase (ESBL) SHV-11 and the carbapenemase KPC-2.

Since community-associated ESBL-producing organisms have been described in Canada and Europe (*7**,**8*), acquisition or in vivo development of ESBL and KPC-producing strains could have occurred outside of the healthcare setting. More likely, the patient acquired her almost completely resistant gram-negative organism in the rural hospital or her local nursing home. To our knowledge, no other clinical isolates with the same antimicrobial phenotype have been seen in patients in either setting before or after the patient's admission. An unsuspected reservoir of patients colonized with antimicrobial drug–resistant gram-negative organisms may exist (*9*). Ideally, an epidemiologic investigation at both the hospital and nursing home would have been performed, but facilities for an investigation involving use of selective microbiologic media and assessment of gastrointestinal carriage of resistant organisms are not typically available in a rural setting. Indeed, most rural hospitals do not even use routine diagnostic tests for detecting resistant gram-negative organisms such as ESBL producers (*10*).

Although much attention has been focused on the progression of antimicrobial drug resistance in gram-positive organisms, the development of alternative antimicrobial agents such as linezolid and daptomycin may mitigate the disastrous scenario of complete resistance to all commercially available antimicrobial agents. However, few drugs are active against multidrug-resistant gram-negative pathogens, and enhanced measures are needed to prevent spread of these organisms. A greater understanding of the modes of spread and acquisition of these organisms is essential for effective control of this problem. We have reported just 1 case of infection with an almost completely resistant gram-negative organism. This case expands the known geographic spread of organisms with this resistance problem. This case also underscores the importance of studying the epidemiology of antimicrobial drug resistance in gram-negative organisms in the rural setting as well as in large metropolitan centers. Dissemination of knowledge regarding appropriate antimicrobial drug susceptibility testing for resistant organisms is also needed.
